# Gastrointestinal Motility, Muscle Relaxation, Antipyretic and Acute Toxicity Screening of Amyrin Type Triterpenoid (Daturaolone) Isolated From *Datura metel* Linnaeus (Angel’s Trumpet) Fruits

**DOI:** 10.3389/fphar.2020.544794

**Published:** 2020-09-25

**Authors:** Saud Bawazeer, Abdur Rauf, Sami Bawazeer

**Affiliations:** ^1^Department of Pharmaceutical Chemistry, Faculty of Pharmacy, Umm Al-Qura University, Makkah, Saudi Arabia; ^2^Department of Chemistry, University of Swabi, Swabi, Pakistan; ^3^Department of Pharmacognosy, Faculty of Pharmacy, Umm Al-Qura University, Makkah, Saudi Arabia

**Keywords:** *Datura metel*, daturaolone, gastrointestinal motility, antipyretic activity, muscle relaxation activity

## Abstract

*Datura metel* Linn is used traditionally for the treatment of various diseases including relaxation of smooth muscles, relief of fever, as well as gastrointestinal disorder. This study deals with the bio-guided isolation of an active, amyrin-type triterpenoid, namely 3-oxo-6-*β*-hydroxy-*β*-amyrin (daturaolone; **1**), from the chloroform fraction of *Datura metel* L. (Angel’s trumpet) fruits and its gastrointestinal motility, antipyretic, and muscle relaxation effects in animal models. The chemical structure of daturaolone (**1**) was elucidated by NMR spectroscopy and crystallography techniques. The chloroform fraction and daturaolone (**1**) were assessed for the GIT motility test. Data exhibited in charcoal meal GI transit test show that chloroform fraction and daturaolone (**1**) significantly reduce GIT motility and increased intestinal transit time, comparable to the standard (atropine), a muscarinic receptor blocking agent. Muscle relaxant potency of the extract and daturaolone (**1**) was assessed in various animal paradigms. In the inclined plane screening test, it produced a significant (P < 0.05) muscle relaxation potential in a dose-dependent manner after 30, 60, and 90 min. Likewise, the muscle relaxation potential of the extract and daturaolone (**1**) was strongly complemented by the chimney and traction test, representing a dominant effect after 60 min of sample administration. The chloroform fraction showed good antipyretic activity, and while daturaolone (**1**) exhibited significant activity at a higher dose, the maximum effect (84.64%) was at 20 mg/kg i.p. In acute toxicity screening test, the chloroform extract (100, 250, 500, and 1,000 mg/kg) and daturaolone (**1**) (5, 10, 20, and 50 mg/kg) were found safe. In conclusion, the chloroform extract and daturaolone (**1**) exhibited strong gastrointestinal motility, muscle relaxation, and antipyretic activity in different animal models and intestinally, was found safe at higher tested doses.

## Introduction

*Datura metel* is a member of the family Solanaceae and is an erect shrub with spreading branches. The plant has a local name in Bengali called Dhutura. *D. metel* grows throughout the year and is commonly known as Devil’s Trumpet in English (Duke and Ayensu, 1985; Dabur et al., 2004). It grows in both hot and humid climate. The plant also has an American origin and is widely distributed in Pakistan, India, South America, and Africa (Sangwan and Camefort, 1983). It has long flowers having purple and white colors which scented up to 6″ (Okwu and Igara, 2009), while the leaves are broad in shape and dark green in color. *D. metel* is 10–20 cm long and 5–18 cm broad. Its fruits are spiny capsules in nature with a thickness of 4–10 cm. *D. metel* is widely used in Bangladeshi herbal medicine (Monira and Munan, 2012). In Chinese traditional system, its flowers are known as baimantuoluo which are used for the treatment of psoriasis and inflammation of the skin (Wang et al., 2008; Monira and Munan, 2012). In Ayurvedic medicinal systems, its seeds are used to cure ulcers, jaundice, diabetes, skin rashes, and bronchitis (Monira and Munan, 2012). Further to these uses, its flowers are involved in cigarette production, while the seeds are used in making tea with potent sedative action in Brazil (Agra et al., 2007). It is also used for the treatment of heart disease, epilepsy, diabetes, insanity, fever, diarrhea, asthmatic anesthetic, hallucinogenic, anti-asthmatic, hypnotic, antitussive, bronchodilator, anodyne narcotic, and skin diseases (Chopra et al., 1956; Chopra et al., 1986; Monira and Munan, 2012). The bioactivities of *D. metel* are due to secondary metabolites present in it. *D. metel* is well documented to contain varieties of potent secondary metabolites including alkaloids, steroids, saponins, flavonoids, triterpenoids, and tannins (Okwu and Igara, 2009). Various types of alkaloids have been detected in different parts of *D. metel*; the numbers of alkaloids increase with the age of the plant (Afsharypuor et al., 1995; Monira and Munan, 2012). The important compounds of *D. metel* are tropane alkaloids including hyoscine, hyoscyamine, littorine, valtropine, acetoxytropine, fastusine, and various withanolides as well as a number of trigloyl ester of tropine (Afsharypuor et al., 1995; Monira and Munan, 2012). An alkaloid known as scopolamine, a major compound isolated from *D. metel*, has been documented for curing various diseases including bronchitis and asthma (Dabur et al., 2004). Other tropane alkaloids isolated from *D. metel* are used as antispasmodic, mydriatic, and sedative agents (Nuhu, 2002). It is also an important source of withanolides, which is used to cure pain and has hallucinogenic potency (Abubakar et al., 2009; Yang et al., 2014; Arjun et al., 2015). The seeds of *D. metel* are used to relieve dental pain. Also, it has wide applications in ayurvedic medication such as the ability to cure hair fall and other skin-related infectious diseases (Soni et al., 2012). The plant extracts of *D. metel* have been documented for antimicrobial and anti-inflammatory properties (Akharaiyi, 2011; Alabri et al., 2014). In this context, atropine isolated from the title plant has been used to dilate the pupil, and it helps in the surgery of the eyes (Satyavati and Raina, 1977). The current study deals with the isolation of a triterpenoid, namely daturaolone (**1**), and the *in vivo* gastrointestinal motility, antipyretic, and muscle relaxation as well as acute toxicity study of the chloroform extract and the isolated daturaolone (**1**).

## Material and Methods

### Plant Material Collection

Fruits of *Datura metel* (Angel’s trumpet) were obtained from the ground of Razagram (Khall, Distt; Dir, KPK, Pakistan. The plant specimen was identified by Dr. Muhammad Ilyas, Head of the Department of Botany, University of Swabi, KPK, Pakistan. The voucher specimen no. Bot (UOS-521) was deposited at the herbarium of the Botany Department, University of Swabi.

### Extractions, Fractionation, and Isolation

**Figure 1:**
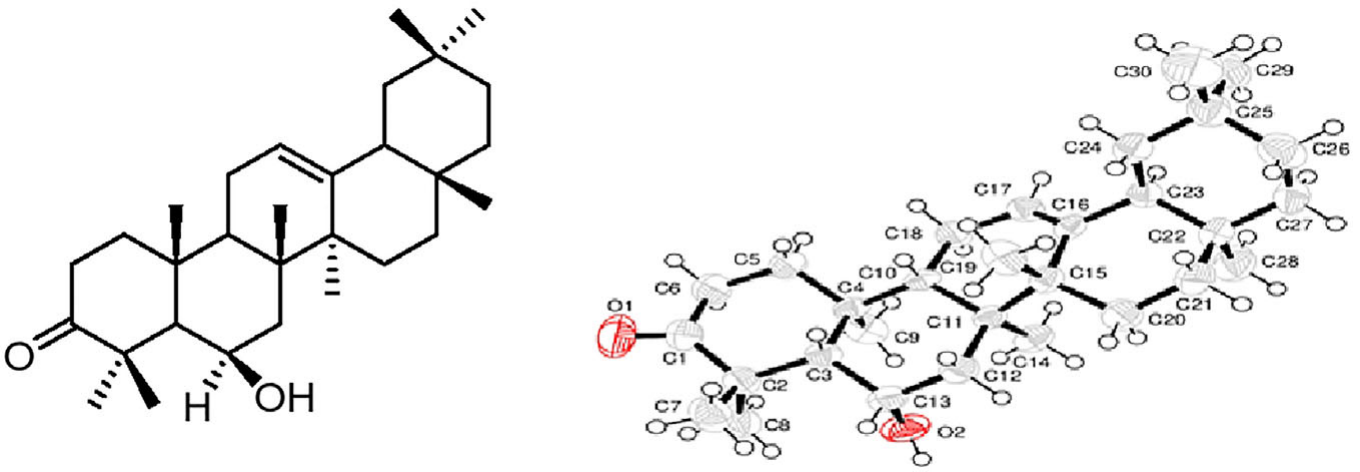
Chemical structure and x-rays’ crystallographic image of daturaolone (**1**) isolated from *Datura metel*.

The fruits (8.2 kg) were dried in a shade at room temperature, ground into powder and then subjected to cold extraction with methanol (×3), which yield 349 g of reddish extract. The extract was suspended in distilled water and then successively extracted with *n*-hexane (52.8 g), chloroform (93.6 g), and ethyl acetate (45.1 g) as per reported methods (Bawazeer et al., 2019). Based on the TLC profile, the chloroform extract (25.0 g) was chromatographed on the silica gel normal phase column. The column was eluted with a mixture of *n*-hexane and ethyl acetate (0:100) of increasing polarity order. One hundred sub-fractions were obtained, which were combined to 12 major sub-fractions (SB-1–SB-12), as per the TLC profile. Based on the TLC profile, SB-7 was subjected to frequent pencil column chromatography (CC), eluting with *n*-hexane and ethyl acetate (14:86), which yielded white crystals. The obtained crystals were washed with *n*-hexane to afford daturaolone (**1**) (2.7 g; 99.87% pure). The chemical structure of daturaolone (**1**) was elucidated by advanced spectroscopic technique. The structure was confirmed by crystallography technique (**Figure 1**), and the spectral data were compared with the reported ones (Wang et al., 2005).

### Animals

Balb/c mice of either sex, weighing 18–22 g were used throughout this work. These animals were placed under standard laboratory conditions. All animals were served with standard laboratory diet and *ad libitum* water. Experiment was performed at the Pharmacy Department University of Swabi as per standard procedure. Studies involving human participants were reviewed and approved by the ethical committee UOS/Pharm-649, Department of Pharmacy, University of Swabi, Swabi, Pakistan.

### Gastrointestinal Motility Test

The chloroform fraction and daturaolone (**1**) were assessed for gastrointestinal motility screening test. In these tests, mice were divided into six groups (*n = 6*). The 1^st^ group received normal saline (10 ml/kg) and acted as a negative control; the 2^nd^ group was administered with the reference drug (atropine; 10 mg/kg, i.p.). The remaining groups were treated with chloroform fraction at the doses 10, 25, 50, 100, and 200 mg/kg, i.p. and daturaolone (**1**) at the doses 2.5, 5, 10, 20, and 40 mg/kg, i.p. After 15 min of treatment, every animal received a 0.3 ml charcoal meal in the form of a suspension in water with 10% vegetable charcoal and 10% gum acacia. Exactly after 30 min of the above administration, all animals were sacrificed by cervical dislocation, and the whole small intestine was detached. Experiments were performed in triplicate, and the percent activity was measured from the distance traveled by charcoal in the small intestine as per our previous methods (Rauf et al., 2016a; Ahmad et al., 2018).

### Muscle Relaxation Assay

The muscle relaxation activity of the chloroform fraction and daturaolone (**1**) was performed using the chimney and inclined plane model according to the standard method (Rauf et al., 2018).

#### Chimney Model

Muscle relaxation potency of chloroform fractions and daturaolone (**1**) was performed according to our reported procedure (Rauf et al., 2018). In this model, a pyrex tube having a diameter of 3 cm and length of 30 cm was used. The design tube was marked 22 cm from the base, and the treated mice were allowed to muscle relaxant traits after 30, 60, and 90 min. The animals were divided into various groups (*n = 6*). Animals in one group were fed with distilled water (10 ml/kg), one group with diazepam (1 mg/kg) as a standard drug, and the remaining animals with chloroform fractions at the doses 10, 25, 50, 100, and 200 mg/kg, i.p. and daturaolone (**1**) at doses 2.5, 5, 10, 20, and 40 mg/kg, i.p. All groups of treated animals were allowed at the one adjacent of the tube and then permitted to move rising from the base to the marked position at 20 cm. When the animals reached the mark then we directly converted the tube to vertical. Those animals which fail to climb to the 20 cm mark of the glass tube in 30 s were counted as animals with muscle relaxation potential.

#### Traction Model

The muscle relaxation effect of chloroform fractions and daturaolone (**1**) was performed according to our reported traction methods (Rauf et al., 2018). These methods were designed by using a metallic wire coated with rubber, and both sides of the wire were connected with a stand around 60 cm upstairs of the laboratory bench. Different groups of animals (*n = 6*) were fed with distilled water (10 ml/kg), diazepam (1 mg/kg), and the chloroform extract and isolated daturaolone (**1**) (2.5, 5, 10, 20 mg/kg i.p.). Then all the animals were open to traction testing after 30, 60, and 90 min. All groups of animals were suspended by back legs to the attached wire then hanging time was recorded for less than 5 s. Failure of each animal to hang to the wire indicated muscle relaxant potency. The activity was performed again and again to calculate the muscle relaxant potency according to reported methods (Rauf et al., 2018).

#### Inclined Plane Model

This model was also used to check the muscle relaxation effect of chloroform fractions and daturaolone (**1**) as per the published method (Rauf et al., 2018). The inclined plane used in this method consisted of two plywood boards which were joined in such a pattern that one plywood board was from the base and the other with the base at 65°. Animals were divided into various groups (*n = 6*); each group was administered with distilled water (10 ml/kg), standard drug (1 mg/kg), chloroform fraction at the doses 10, 25, 50, 100, and 200 mg/kg, i.p., and daturaolone (**1**) at the doses 2.5, 5, 10, 20, 40 mg/kg, i.p. After 30, 60, and 90 min of the administration, animals were permitted on the higher portion of the inclined plane for 30 s to hang or fall.

#### Yeast Induced Antipyretic Activity

The antipyretic effect of chloroform extract and daturaolone (**1**) isolated from the title plant was evaluated using a standard procedure (Muhammad et al., 2012). Animals were distributed into various groups (*n = 6*). All groups of animals were subjected to fasting and allowed free access to drinking water. Among the divided groups, Group I received saline (control), Group II received the standard drug (Paracetamol), while the remaining groups (III–VII) received the fraction at different doses 10, 25, 50, 100, and 200 mg/kg, and daturaolone (**1**) at 2.5, at 10, 15, and 20 mg/kg. The normal temperature was noted with the help of a numerical thermometer, and then pyrexia was induced in all groups of animals by introducing aqueous suspension (20%) of Brewer’s yeast (10 ml/kg, s.c.). After 24 h, the rectal temperature was noted and groups (III–VII) were administered with the above doses. After drug administration, the rectal temperature was once more noted periodically at 1, 2, 3, 4, and 5 h of drug administration. The percent effect was calculated using our previous formula (Muhammad et al., 2013).

### Toxicological Screening

The acute toxic effect of chloroform fraction and daturaolone (**1**) was performed in mice of both sexes as per our standard method (Rauf et al., 2016a). To find the toxicological profile of the extract and daturaolone (**1**), mice were starved for 16 h earlier to the experiment. All animals were divided into various groups (*n = 6*). Group I (Control group) was fed with normal saline (10 ml/kg). The remaining animals were treated with chloroform fraction in various doses (50, 100, 250, 500, and 1,000 mg/kg, (p.o.) and daturaolone (**1**) at (10, 25, 50, 100, 250, 500 mg/kg, (p.o.). Treated mice were then allowed free access to water and food and were noted for gross behavior change as well as mortality for 24 h.

### Statistical Analysis

All results of this biological screening are presented as the mean ± standard error of the mean (SEM). To find the level of significant differences (*p* < 0.05 or 0.01) among the experimental groups, One-way analysis of variance (ANOVA) was achieved by Dunnett’s multiple comparison test.

## Results and Discussion

### Structure Elucidation

Daturaolone (**1**) was isolated from chloroform fraction and identified as 3-oxo-6*β*-hydroxy-*β*-amyrin by different spectroscopic techniques.

Daturaolone (**1**) was isolated as white crystals; IR (KBr, cm^−1^) ν_max_ = 1599 (C=C), 1699 (C=O), 2,918 (C-H), 3650 (OH). ^1^H NMR (500 MHz, CDCl_3_): *δ* 0.83 (s, 3H, CH_3_-28), 0.85 (s, 6H, CH_3_-29, CH_3_-30), 1.08 (s, 3H, CH_3_-27), 1.15 (s, 3H, CH_3_-23), 1.22 (t, 2H, CH_2_-22), 1.32 (s, 3H, CH_3_-26), 1.40 (s, 3H, CH_3_-24), 1.49 (s, 3H, CH_3_-25), 1.53 (s, 2H, CH_2_-7), 1.62 (t, 2H, CH_2_-15), 1.64 (d, 1H, CH-5), 1.65 (d, 2H, CH_2_-19), 1.67 (t, 1H, CH-18), 1.76 (m, 2H, CH_2_-11), 1.98 (t, 2H, CH_2_-16), 2.06 (t, 1H, CH-5), 2.22 (3, 2H, CH_2_-1), 2.73 (3, 2H, CH_2_-2), 4.49 (brs, 1H, CH-6), 5.24 (s, 1H, CH_2_-12); ^13^C NMR (125 Hz, CDCl_3_): *δ* 16.5 (CH_3_-19), (CH_3_-19), 18.6 (CH_3_-26), 23.6 (CH_3_-11, 24), 23.9 (CH_3_-30), 25.8 (CH_3_-23), 25.9 (CH_3_-27), 26.1 (CH_2_-15), 26.9 (CH_2_-16), 28.3 (CH_3_-28), 31.0 (C-20), 32.8 (C-17), 33.3 (CH_3_-29), 34.0 (CH_2_-21), 34.4 (CH_2_-2), 36.3 (C-10), 37.0 (CH_2_-22), 39.0 (C-14), 40.6 (CH_2_-1), 41.6 (CH_2_-7), 42.5 (C-8), 46.7 (CH_2_-19), 47.3 (CH-9, 18), 48.7 (C-4), 56.5 (CH-5), 69.3 (CH-6), 121.2 (CH-12), 144.5 (C-13), 216.6 (C-3), ppm; HRMS (ESI) *m/z*: calcd. for C_30_H_48_O_2_ [M]^+^ 440.3710, found 440.3700. The spectroscopic data were compared to those published in the literature for the known compound and were found identical (Wang et al., 2005; Rauf et al., 2016b); furthermore, the daturaolone (**1**) was confirmed by X-ray crystallography data (**Figure 1**).

### Effect on GIT Motility

**Figure 2:**
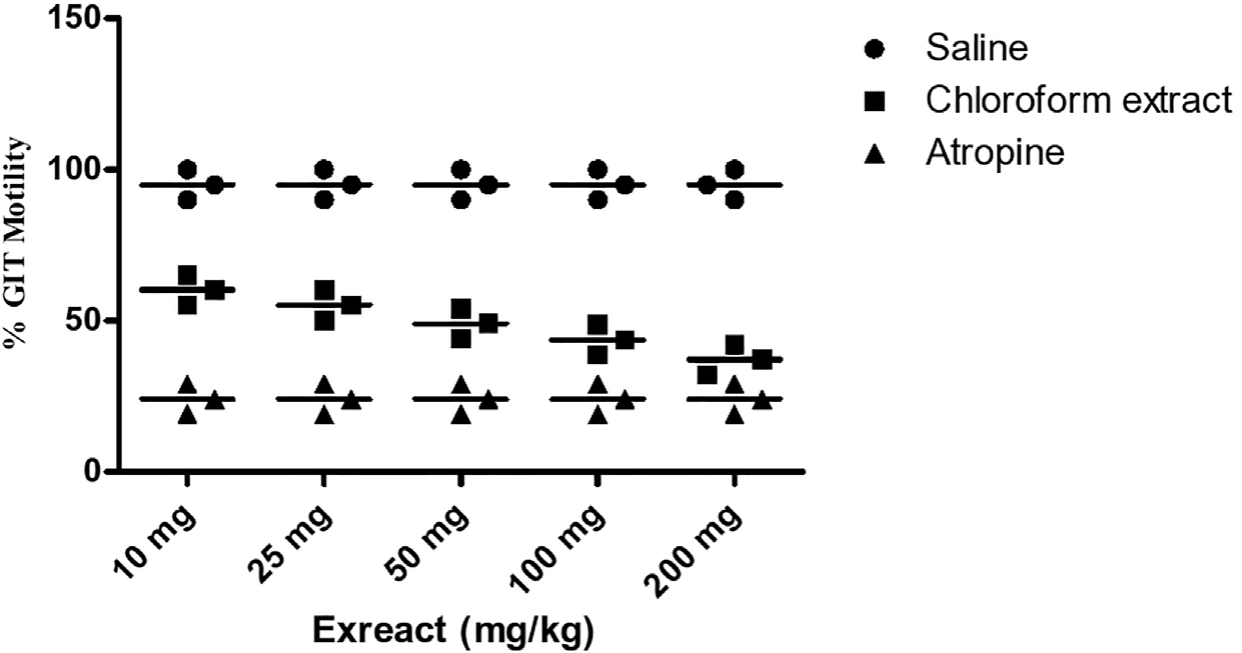
Bar diagram indicated the GIT motility profile of chloroform extract in a dose-dependent manner of charcoal meals through small intestine of mice.

**Figure 3:**
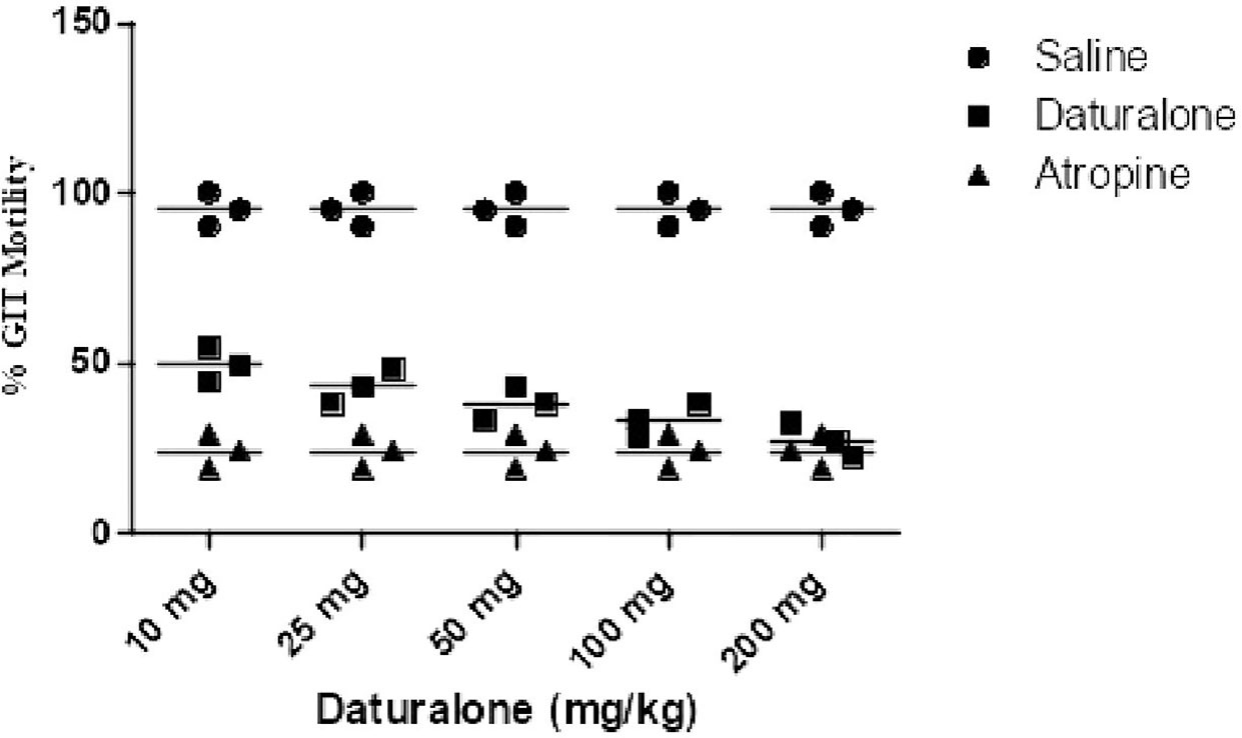
Bar diagram indicated the GIT motility screening profile of daturalone (**1**) in dose-dependent method of charcoal meals through the small intestine of mice.

Results of the GIT motility test of the extract and daturaolone (**1**) are given in **Figures 2**, **3**. A significant movement of the charcoal meal was recorded in the intestine of mice due to chloroform at a dose of 200 mg/kg; similarly, daturaolone (**1**) exhibited excellent movement of the charcoal meal at a dose of 20 mg/kg. The normal saline was used as a positive control. The tested fraction attenuated the movement of charcoal 60% as compared to normal saline, and the positive control demonstrated 24% motility (**Figure 2**). Multiple comparison screening tests indicated that the extract and daturaolone (**1**) at a dose of 200 and 20 mg/kg significantly reduce the GIT motility (**Figure 3**).

### Muscle Relaxant Effect

In both muscle relaxant testing paradigms, the chloroform fraction and tested daturaolone (**1**) demonstrated a significant muscle relaxant effect (**Tables 1**, **2**). Dose- and time-dependent effects were noticed. The effect of chloroform fraction was 49.32% in both tested experimental paradigms. The muscle coordination activity of daturaolone (**1**) was greater than that of chloroform fraction. The isolated daturaolone (**1**) also demonstrated a significant dose- and time-dependent effect. However, the effect of daturaolone (**1**) was highly significant from the start of the experiment as compared to the chloroform fraction.

**Table 1:** Effect of chloroform fraction and daturaolone (**1**) on relaxation of muscles.

Group	Dose/kg	Chimney test			Traction test		
		30 min	60 min	90 min	30 min	60 min	90 min
Distilled water	10 ml	0.0 ± 0.00	0.0 ± 0.00	0.0 ± 0.00	0.0 ± 0.00	0.0 ± 0.00	0.0 ± 0.00
Diazepam	0.25 mg	100.0 ± 0.0***	100.0 ± 0.0***	100.0 ± 0.0***	100.0 ± 0.0***	100.0 ± 0.0***	100.0 ± 0.0***
Chloroform	10	3.2.64 ± 1.40	9.87 ± 1.28	14.89 ± 1.20	1.99 ± 1.88	5.10 ± 1.70	9.43 ± 1.50
	25	07.09 ± 1.60	12.65 ± 1.66	17.98 ± 1.88	4.8.97 ± 2.00	9.10 ± 1.98	15.00 ± 1.80
	50	12.99 ± 1.99	18.33 ± 1.66	23.45 ± 1.41	9.2.34 ± 2.88	14.98 ± 2.9	19.22 ± 2.00
	100	20.10 ± 1.18	26.35 ± 1.87	31.98 ± 1.98	17.43 ± 1.88	22.12 ± 191	27.44 ± 1.65
	200	42.54 ± 2.00**	49.32 ± 2.12**	55.09 ± 2.19***	33.66 ± 189**	38.98 ± 2.55**	41.23 ± 2.12***
Daturaolone (**1**)	2.5	4.44 ± 1.55	10.981.87	17.98 ± 1.98	2.12 ± 1.44	7.7.60 ± 1.90	11.12 ± 2.00
	5	9.23 ± 1.80	15.12 ± 1.34	21.77 ± 1.98	5.7.23 ± 1.60	11.99 ± 1.20	18.01 ± 1.30
	10	15.23 ± 2.08	22.98 ± 2.11	27.98 ± 1.98	11.99 ± 2.16	17.09 ± 2.28	24.07 ± 2.20
	15	26.91 ± 1.44	31.02 ± 1.56	37.20 ± 1.70	20.09 ± 1.76	27.00 ± 1.63	35.99 ± 1.59
	20	53.88 ± 1.28**	59.98 ± 1.98***	66.32 ± 1.99***	44.10 ± 1.87**	51.16 ± 1.98***	58.07 ± 2.03***

The data of muscle relaxant effect represented the % activity of mice (n = 6) in both models at 30, 60, and 90 min after administration of samples. The data is represented as the mean ± SEM (n = 6); *p < 0.05, **p < 0.0.01, ***p < 0.001, all values as related with control.

**Table 2:** Effect of chloroform fraction and daturaolone (**1**) on muscle relaxation (Inclined plane test).

Group	Dose/kg	Inclined plane test		
		30 min	60 min	90 min
Distilled water	10 ml	0. ± 0.00	0. ± 0.00	0. ± 0.00
Diazepam	0.25 mg	100. ± 0.00***	100. ± 0.00***	100. ± 0.00***
Chloroform	10	2.9. 33 ± 1.49	8.83 ± 1.99	14.00 ± 1.80
	25	8.1.02± 1.60	13.60 ± 1.68	19.92 ± 1.98
	50	16.09± 1.90	20.30 ± 1.80	26.43 ± 1.40
	100	25.98± 1.19	30.30 ± 1.82	37.91 ± 1.90
	200	33.32 ± 2.07*	38.32 ± 2.12**	43.11. ± 2.00**
Daturaolone (**1**)	2.5	3.23.45 ± 1.50	8.2.96.86	15.98 ± 1.92
	5	7.88 ± 1.81	12.10 ± 1.32	17.78 ± 1.96
	10	13.25 ± 2.03	18.90 ± 2.15	13.91 ± 1.90
	15	24.94 ± 1.45	29.09 ± 1.57	35.30 ± 1.79
	20	49.80 ± 1.29**	54.91 ± 1.56***	62.21 ± 1.22***

The data of muscle relaxant effect represented the % activity of mice (n = 6) in both models, at 30, 60, and 90 min after administration of samples. The data is represented as the mean ± SEM (n = 6); *p < 0.05, **p < 0.0.01, ***p < 0.001; all values as related with control.

### Antipyretic Effect

**Figure 4:**
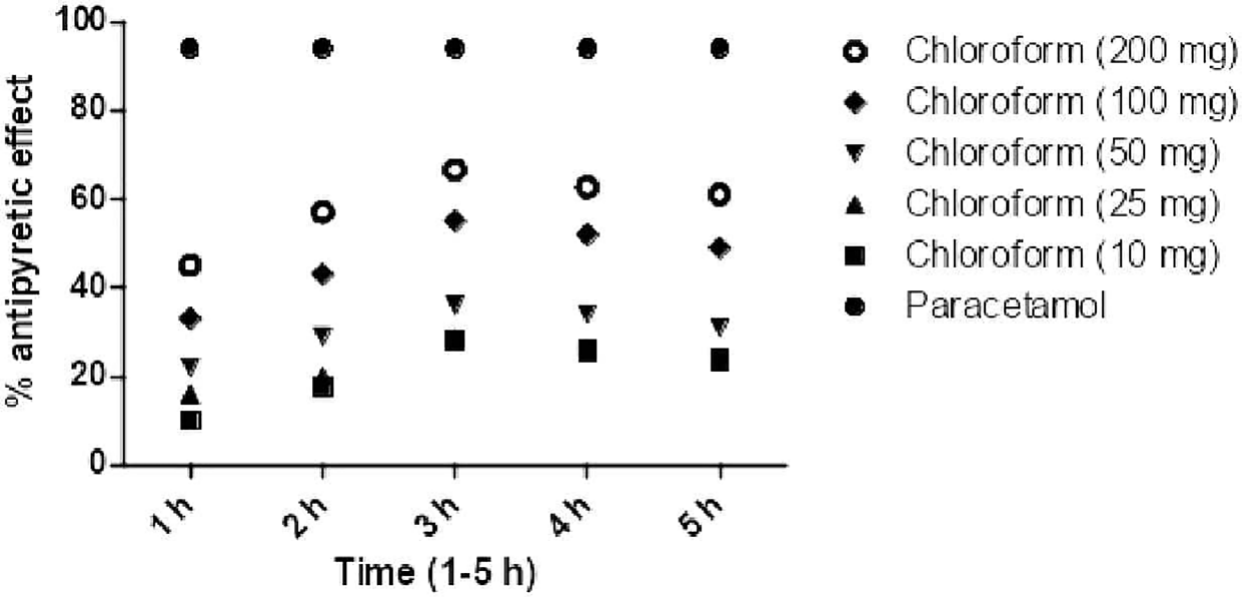
Percent antipyretic effects of chloroform extract in yeast induced test at various doses.

**Figure 5:**
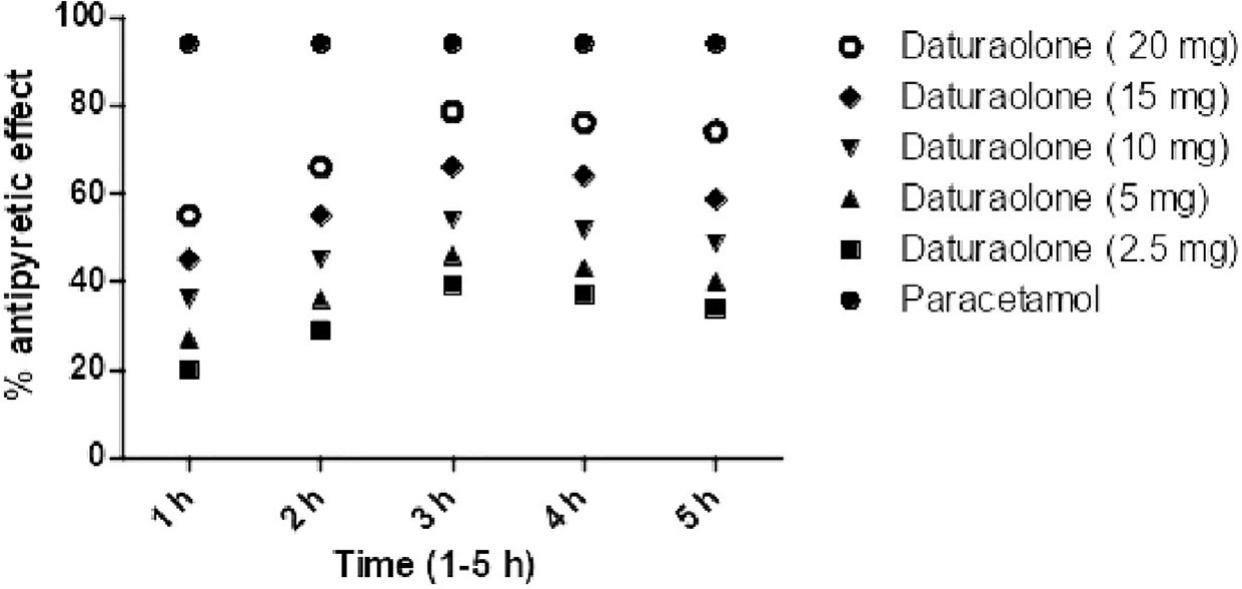
Percent antipyretic effects of daturaolone (**1**) in yeast-induced test at various doses. All values are represented as the mean ± SEM for all groups of six animals.

Post treatment of chloroform fraction and daturaolone (**1**) showed a significant (p < 0.001) antipyretic activity in febrile mice during different assessment times (1–5 h). The promising antihyperthermia activity (66.98% was recorded for chloroform fraction at 200 mg/kg i.p., while for daturaolone (**1**) was (78.98%) at 20 mg/kg i.p. after 3 h of sample administration (**Figures 4**, **5**).

### Acute Toxicity

The acute toxicity of chloroform fraction was screen in doses, 100, 250, 500, and 1,000 mg/kg. During 24 h assessment no toxicity was observed. Similarly, daturaolone (**1**) in doses of, 5, 10, 20, and 50 mg/kg showed no mortality during 24 h of assessment. Neither gross behavior changes nor mortality was observed for the extract and daturaolone (**1**).

Pretreatment with chloroform fractions and daturaolone (**1**) caused marked (P < 0.05) dose-dependent decrease in distance traveled by charcoal meal and showed a remarkable increase in GI transit time. The percent activity exhibited significant effect of extract and daturaolone (**1**) isolated from the title plant. When investigated in charcoal meal gastrointestinal transit, the extract and daturaolone (**1**) caused a dose-dependent decrease in the propulsive association of charcoal and displayed strong GIT motility effects as compared to atropine sulfate, a muscarinic receptor blocking agent. It has been reported that blockage of muscarinic receptors in the GIT has a promising effect on the GIT smooth muscle motility. Normally, tone and propulsive moments are diminished, thus causing an increase in the intestinal transit time (Rauf et al., 2016a). Consequently, it is recommended that anti-GIT motility of chloroform fraction and daturaolone (**1**) may be due to the blockage of muscarinic receptors in the GIT. In short, the chloroform fraction and daturaolone (**1**) isolated from title plant provoked a strong decrease in GIT motility with significant safety in initial studies. More studies are required to ascertain clinical utility.

*In vivo* traction test, chimney test, and inclined plane screening test are the most widely used tools for evaluation of muscle relaxant activity (Rauf et al., 2014). Results of the current finding indicated a strong potential of title plant fraction and daturaolone (**1**) for muscle relaxation. The muscle relaxation effect of chloroform fractions and daturaolone (**1**) isolated from the title plant is displayed in **Table 1** and **Figure 1**. The tested fraction and isolated daturaolone (**1**) exhibited marked muscle relaxation in several animal models such as traction test, chimney test, and inclined plane model. Muscle relaxation effect of the extract and isolated daturaolone (**1**) was assessed in several muscle relaxant paradigms including traction test, chimney test, and inclined plan model. In these models, the muscle relaxant efficiency was assessed after 30, 60, and 90 min of treatment of chloroform fractions and daturaolone (**1**). Significant activity was observed after 60 min of chloroform fractions and daturaolone (**1**) administration. In all tested model the maximum muscle relaxation was noted at a higher dose. It is concluded that chloroform extract and daturaolone (**1**) exhibited promising muscle relaxation effects in various *in vivo* protocols. The hypodermic injection of brewer’s yeast induced pyrexia by eventually increasing the synthesis of prostaglandin, and this method is considered the most important *in vivo* analysis test for the evaluation of antipyretic activity (Bertram, 2007; Rauf et al., 2014). Yeast-induced pyrexia is known as none pathogenic fever, the etiology of which is the making of prostaglandin (PG). Inhibition of PG synthesis may be the potential mechanism of antipyretic potency; that of paracetamol the inhibition of prostaglandin can be attained by blocking the cyclooxygenase enzyme effect (Rauf et al., 2014; Barkatullah et al., 2016). The promising antipyretic effect of chloroform fractions and daturaolone (**1**) is associated with numerous mediators based on pyrexia, mainly prostaglandins (Islam et al., 2015; Barkatullah et al., 2016). The intraperitoneal administration of chloroform fractions and daturaolone (**1**) remarkably attenuated the rectal temperature of yeast-induced febrile mice. Therefore, it can be concluded that chloroform fractions and daturaolone (**1**) interface with the synthesis/release of PG at any stage. We recommend chloroform extract and daturaolone (**1**) to researchers for further detailed and careful studies as potential plants for the controlling of various diseases. The promising gastrointestinal motility, muscle relaxation, antipyretic effects of chloroform fractions and isolated daturaolone (**1**) provide a strong scientific background to the traditional uses of *Datura metel* in the treatment of fever and muscle relaxation.

## Conclusions

Phytochemicals can afford an excellent pharmacophore template for novel drug discovery which is used for the cure of many diseases. The crude extract/fraction and the isolated compound, daturaolone (**1**) exhibited significant gastrointestinal motility cessation, muscle relaxation, antipyretic effects. The fractions and daturaolone (**1**) were found safe when tested in a wide range of doses for acute toxicity. However, its safety and effect should further be assessed in chronic disease animal models. Thus daturaolone (**1**) could be used as lead compound for new drug development for fever, muscle relaxation, and associated gastrointestinal problems.

## Data Availability Statement

The original contributions presented in the study are included in the article/supplementary material; further inquiries can be directed to the corresponding author.

## Ethics Statement

The studies involving human participants were reviewed and approved by ethical committee UOS/Pharm-649, Department of Pharmacy, University of Swabi, Swabi, Pakistan.

## Author Contributions

SauB supervised this project. AR and SamB were involved in the experimental part and writing of this paper. All authors contributed to the article and approved the submitted version.

## Funding

The work is funded by grant number 14-MED333-10 from the National Science, Technology and Innovation Plan (MAARIFAH), the King Abdul-Aziz City for Science and Technology (KACST), Kingdom of Saudi Arabia.

## Conflict of Interest

The authors declare that the research was conducted in the absence of any commercial or financial relationships that could be construed as a potential conflict of interest.
